# *ACADVL* Deep Sequencing in a Case Study: Beyond the Common c.848T>C Pathogenic Variant

**DOI:** 10.3390/genes16050538

**Published:** 2025-04-30

**Authors:** Francesco Baldo, Luisa Zupin, Andrea Magnolato, Valeria Capaci, Maria Teresa Bonati

**Affiliations:** 1 Institute for Maternal and Child Health IRCCS Burlo Garofolo, 34137 Trieste, Italy; 2 Department of Medicine, Surgery and Health Sciences, University of Trieste, 34100 Trieste, Italy

**Keywords:** metabolic newborn screening, plasma acetylcarnitine profile, white blood cell VLCAD assay, *ACADVL*, synonymous hypomorphic variants, gene expression

## Abstract

**Background:** Very-long-chain acyl-CoA dehydrogenase deficiency (VLCADD) is caused by biallelic pathogenic variants in *ACADVL* (acyl-CoA dehydrogenase very-long-chain), leading to impaired fatty acid oxidation and the accumulation of long-chain acylcarnitine. We report a single case of a two-year-old girl, whose neonatal metabolic screening revealed an acylcarnitine profile suggestive of VLCADD, with residual enzymatic activity of 19.8%. **Methods:** We performed *ACADVL* whole-gene sequencing. We then carried out an in silico analysis of the potential effects of the variants with dedicated tools, assessing splicing, RNA structure, RNA binding factors, and protein structure. We also conducted gene expression analysis. **Results:** Genetic testing identified her as compound heterozygous for the pathogenic *ACADVL* variant (NM_000018.3):c.848T>C, inherited from her mother, and for the two paternal variants, c.-64T>C in the basal promoter and c.957G>A, a synonymous substitution in exon 10. Gene expression analysis revealed reduced *ACADVL* mRNA levels in the proband’s blood cells but without abnormal isoform production. A decreased expression of the paternal allele carrying the 957A was also observed. Despite this significant reduction in mRNA levels, the underlying mechanism remains unclear. **Conclusions:** Although currently healthy, due to the VLCAD residual activity within the range associated with the mild form of the disease, the child might be at potential risk for metabolic decompensation or late-onset VLCADD. Our results indicated an allelic imbalance in mRNA expression and c.957G>A is identified as a hypomorphic allele. This suggests that deep *ACADVL* sequencing is a valuable tool for correlating genetic variants with enzymatic activity levels.

## 1. Introduction

Metabolic newborn screening implementation has revolutionized the diagnosis of inherited metabolic diseases. The screening is based on dried blood spot analysis by using Tandem Mass Spectrometry and can identify several severe inherited metabolic conditions. Consequently, the confirmation of observed alterations is achieved through genetic analysis and the assessment of the enzymatic activity of the affected protein. The majority of the detected diseases presented recessive inheritance [1]. In 2016–2017, in Italy, the nationwide newborn metabolic screening was extended to around 40 conditions, whose list was reported by Ruoppolo et al. in 2022 [2].

Very-long-chain acyl-CoA dehydrogenase deficiency (VLCADD) is one of the conditions included in the newborn Italian screening, and it is caused by biallelic pathogenic variants in *ACADVL* (acyl-CoA dehydrogenase very-long-chain), resulting in the impairment of fatty acid oxidation with the accumulation of long-chain acylcarnitines [3]. Very-long-chain acyl-CoA dehydrogenase (VLCAD) is a mitochondrial enzyme participating in the first step of fatty acid oxidation, oxidizing the β-carbon of fatty acyl-CoAs with 12–24 carbons [4].

Patients affected by VLCADD may present wide clinical heterogeneity with different severity, ranging from neonatal hypertrophic cardiomyopathy, liver failure, and hypoketotic hypoglycemia (severe form with multiorgan failure and poor prognosis) to juvenile recurrent rhabdomyolysis triggered by infection, fasting, or exercise (mild form) [5,6].

A patient’s classification according to this heterogeneity can be based on the level of enzymatic activity. Under 10% residual activity (RA), the disease manifests in the severe neonatal form. At RA of 10–20%, the individuals are at risk of developing symptoms, especially with high energy demands or infective illness. Over 30% of RA individuals are asymptomatic [5,6,7].

The uncertain zone between 10 and 20% of RA is clinically problematic to manage and follow up on the potential risk of developing the disease [7].

The genetic analysis may further complicate the clinical prognosis when only single heterozygous pathogenic variants are identified. While a newborn may simply be a healthy carrier, these variants can be associated with significantly reduced residual enzymatic activity, sometimes overlapping with levels observed in mildly affected patients. Nevertheless, a clear genotype–phenotype correlation has not yet been defined. Moreover, identifying hypomorphic alleles or variants of uncertain significance (VUS) can make the evaluation even more complex [8].

The assay with [9,10-3H]oleic acid as substrate performed in fibroblasts at 37 °C and 41 °C allows us to discriminate true VLCADD patients from healthy individuals [9]. However, the need to obtain a skin biopsy from a healthy newborn makes the test impractical.

Therefore, the integration of enzymatic, genetic, metabolic, and clinical data is essential but sometimes insufficient to support clinicians in determining whether clinical surveillance is warranted and when it should begin [1].

Our study reports a two-year-old girl whose acylcarnitine profiles at neonatal metabolic screening and follow-up visits were suggestive of VLCADD and showed an RA of 19.8%. The initial genetic analysis identified the common c.848T>C pathogenic variant, previously correlated in heterozygosity to an RA of 30–40% [9], thus not explaining the biochemical findings.

Therefore, we performed *ACADVL* whole-gene sequencing, gene expression analysis following the consultations of in silico prediction tools, and a careful review of the gene variants.

## 2. Materials and Methods

### 2.1. Patient Enrollment

The newborn patient, identified as potentially affected by VLCADD or β-oxidation defect through newborn metabolic screening (NBS) results, was initially referred to the Neonatology Department and subsequently evaluated at the Pediatric Clinic of Maternal and Child Health IRCCS Burlo Garofolo (Trieste, Italy) for comprehensive diagnostic assessment.

The Ethical Committee of the IRCCS Burlo Garofolo reviewed and approved the study according to the Declaration of Helsinki. The proband’s parents gave informed written consent.

### 2.2. Newborn Screening and Confirmatory Testing

According to the recommended time frame, NBS samples of dried blood spots (DBSs) were taken 48–72 h after birth. The blood level of tetradecenoyl carnitine (C14:1) was measured by tandem mass spectrometry at the University Hospital of Padua (Italy). Second and third DBSs were requested within 48 h and 7 days upon abnormal first- and second-screening results, respectively [2]. Plasma acylcarnitines, liver enzymes, ammonium, creatine phosphokinase (CPK), and activated partial thromboplastin time ratio (aPTTR) were measured to confirm the diagnosis further.

The Molecular Genetics Laboratory of the University Hospital of Padua performed Next Generation Sequencing (NGS), analyzing all exons and exon/intron boundaries of the *ACADVL* gene using DNA extracted from the proband’s blood.

Concurrently, the Metabolic Diseases Research Unit of the Bambino Gesù Pediatric Hospital in Rome (Italy) conducted a functional study to evaluate residual VLCAD enzymatic activity in the proband’s lymphocytes. The analysis was performed using HPLC-UV [7], with VLCAD activity measured as the rate of palmitoyl-CoA oxidation in the white blood cell.

### 2.3. ACADVL Deep Sequencing

Genomic DNA was extracted from whole blood samples of the proband and her parents using the Puregene Blood Kit (Qiagen, Hilden, Germany). We then amplified the entire *ACADVL* gene (including promoter and intronic regions) by PCR using overlapping primers (available on request) and KAPA Hotstart ReadyMix (KK5601, Roche, Basel, Switzerland). Sanger sequencing was subsequently performed to analyze the amplified products.

Variants interpretation was conducted by using the ClinVar [10] and HGMD professional (version 2025.1, accessed on 21 April 2025) [11] databases.

### 2.4. Protein Molecular Modelling

The three-dimensional structure of the VLCAD protein (PDB ID: 2UXW) was obtained from the Protein Data Bank (https://www.rcsb.org/structure/2UXW, accessed on 21 April 2025). To evaluate the structural impact of the p.Val283Ala mutation, we employed an in silico mutagenesis approach. Using Chimera software (version 1.19) [12], the mutation was introduced into the wild-type protein model. The mutated structure was then superimposed onto the original wild-type configuration, and the Root Mean Square Deviation (RMSD) was calculated to quantify the extent of conformational changes induced by the variant.

### 2.5. ACADVL RNA Evaluation

We conducted RNA in silico analysis with the following tools: TFBIND [13] to investigate the protein factors binding to the promoter region; SplieAI [14] to assess splicing; RNA folds web server [15] to model the secondary structure; and SplieAID [16] to investigate the consensus sequence for RNA binding factors.

We extracted the RNA of the proband and two healthy controls from 1.5 mL of fresh whole blood with the QIAamp RNA Blood Mini Kit (Qiagen, Hilden, Germany) and then retro-transcribed it by using the High-Capacity cDNA Reverse transcription kit (Thermo Fisher Scientific, MA, USA).

Based on the genetic results (see Section 3.2), we quantified two possible *ACADVL* isoforms caused by exon 10 skipping using two primer sets. The first set is composed of a forward primer located at the exon 9–exon 10 junction and a reverse primer within exon 10. The second set is composed of a forward primer spanning the exon 9–exon 11 junction, and a reverse primer located in exon 11 (ex9-ex10_F ATTACCCATGGGCCCCCT, ex9-ex10_R GGATGTGCATGGCAACCTTG, ex9-ex11_F GCATTACCCAGTAGATCATGCC, ex9-ex11_R GCGGCCTCTATCTGGAAGTC). The *H3* gene was used as a calibrator and control (primer fw GTGAAGAAACCTCATCGTTACAGGCCTGGT, primer rev CTGCAAAGCACCAATAGCTGCACTCTGGAA). Following the manufacturer’s instruction, the isoform levels were analyzed at the CFX Opus Biorad instrument with the iTaq Universal SYBR Green Supermix (Biorad, CA, USA).

We performed next-generation sequencing (NGS) to evaluate the relative expression levels of the maternal and paternal alleles. To this end, we amplified complementary DNA (cDNA) by PCR using primers targeting the region encompassing the two variants located in exons 9 and 10 of the ACADVL gene (Fw TGGATCAGTAATGGGGGCCT, Rv AGCCATGCCAAACCTTCCAT, with Illumina NGS adaptors) by using the NEBNext^®^ Ultra™ II Q5^®^ Master Mix (New England Biolabs, MA, USA) on the Miseq Illumina platform (Illumina, CA, USA) and analyzed it with the Galaxy Europe online tool [17].

## 3. Results

### 3.1. Clinical Report

At the last follow-up, the patient, who was 21 months old, exhibited normal psychomotor development and growth (her weight was 10.6 kg, in the 18th centile). The girl had a gastrointestinal infection at the age of one for which she did not require hospitalization.

She was the first child of healthy, unrelated Italian parents of Caucasian ethnicity, born at 42 weeks and 1 day of gestation via emergency cesarean section due to failure to progress in labor, following an uneventful pregnancy conceived through medically assisted procreation with intracytoplasmic sperm injection (ICSI). Her family history was unremarkable. Her birth weight was 36,500 g (50–75th centile), birth length 50.6 cm (50–75th centile), and occipitofrontal circumference 33 cm (10–25th centile). Apgar scores were 9 (1 min) and 10 (5 and 10 min).

On the fourteenth day after birth, she was referred due to the biochemical diagnosis of a β-oxidation defect, in particular of VLCADD, detected through NBS. Indeed, the first DBS showed a significantly elevated C14:1 value (1.28 µmol/L, normal value < 0.40), which decreased in the second (0.48 µmol/L) and third (0.31 µmol/L) DBS. The plasma acylcarnitine profile confirmed an altered pattern, with increased C10 (0.27 µmol/L), C12 (0.34 µmol/L), C12:1 (0.21 µmol/L), and C14:1 (0.42 µmol/L). Biochemical tests also included blood count, CPK, ammonium, and aPTTR dosage, which were average, and AST and ALT, which were just above the upper reference limit (97 U/L and 45 U/L, respectively). The newborn underwent blood samples for diagnostic genetic and enzymatic analyses.

Otoemissions, electrocardiogram, echocardiogram, and abdominal ultrasound, the last of which was also performed at 21 months, were all normal. Patient neurological examination was also standard.

The acylcarnitine profile was reassessed at 14 and 21 months of age, revealing a stable elevation of C12, C12:1, C14, C14:1, C14:2, and C16:1 (Supplementary Table S1). In contrast, AST, ALT, and CPK levels remained within the normal range on both occasions. A blood sample for genetic research analysis was obtained at the 14-month follow-up.

At 21 months of age, annual follow-up was recommended. We advised the parents to exercise particular caution during major infectious episodes—especially gastroenteritis—and to promptly seek care at the emergency department for the initiation of intravenous glucose rehydration when needed.

### 3.2. Genetic and Enzymatic Assessment

In the NGS sequencing, the proband was found to be a heterozygous *ACADVL* variant (NM_000018.3):c.848T>C inherited from the mother, classified as pathogenic in ClinVar and HGMD.

The enzymatic activity of VLCAD protein showed a residual enzymatic activity of 19.8%, which is lower than that typically observed in heterozygous carriers (~30–40%), but it does not fall in the range of severely affected patients (<10%) [5,6,7]. 

Therefore, we performed, on a research basis, a Sanger analysis of the whole gene to investigate if some additional genetic variants were initially overlooked. We detected two additional variants inherited from her father: c.-64T>C in the basal promoter (ClinVar classification: benign, variant ranked in the FDA database, absent in HGMD), and c.957G>A in exon 10 but resulting in a synonymous substitution (ClinVar classification: conflicting classifications of pathogenicity; HGMD classification: possible disease-causing mutation—uncertain/less confident). The c.848T>C was already described by Hesse et al. (2018) [9] in compound heterozygosity with the c.957G>A synonymous variant, resulting in comparable enzymatic activity levels (24%) with those found in our proband. Neither deletions nor insertions were seen (Table 1).

### 3.3. In Silico Characterization of the Variants

Promoter c.-64T>C variant. The c.-64T>C variant is located in the promoter region. Therefore, we investigated the potential alteration of transcription factor binding sites by using the TFBIND tool [13].

**Figure 1:**
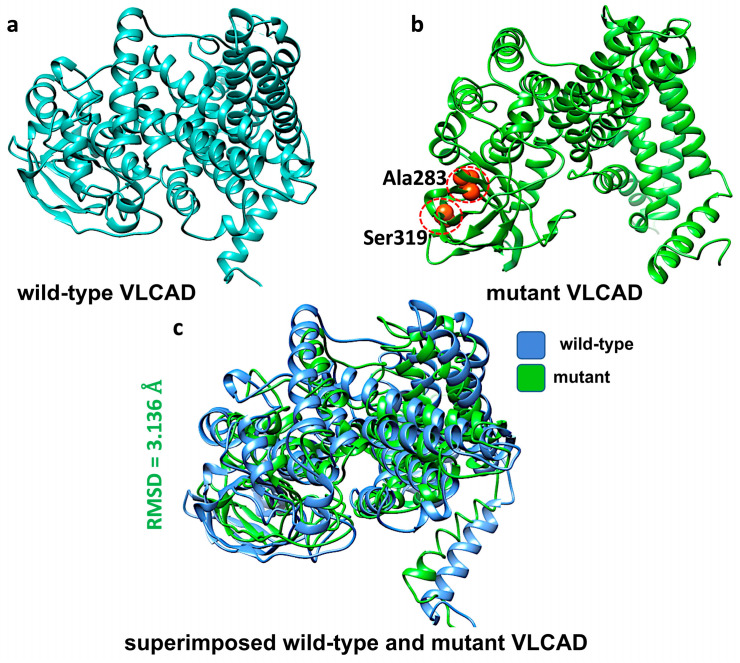
Structural modeling of VLCAD protein in its wild-type and mutant forms and their superposition. (**a**) Predicted VLCAD wild-type structure, (**b**) predicted VLCAD mutant structure with mutation sites (Ser319 and Ala283) highlighted in orange color, and (**c**) superposition of the mutant structure on the wild-type, illustrating structural differences between them as RMSD.

This variant was predicted to disrupt a p300 transcription factor binding site. However, p300, primarily functions as a co-activator of transcription factors or as an acetyltransferase [18] and the region surrounding c.-64T>C already includes two mismatches relative to the p300 consensus motif (GG**GA**GTG mismatches highlighted in bold). All this evidence, along with its classification in ClinVar, supports the interpretation of this variant as benign.

c.848T>C missense variant. This variant is localized in exon 9, causing the substitution of valine with alanine at position 283 (p.Val283Ala). Although not situated within a known binding or active site of the protein, the affected residue lies in an outer loop region, which may influence the protein’s 3d structure. Using Chimera software, we introduced the Val283Ala mutation into the VLCAD protein structure (Figure 1a,b). We aligned the mutant model with the wild-type structure and measured the Root Mean Square Deviation (RMSD) to evaluate how this mutation influenced the protein’s conformation. We observed a structural alteration between the wild-type and mutant forms, with an RMSD value of 3.136 Å (Figure 1c).

c. 957G>A synonymous variant. The c.957G>A synonymous variant is localized in exon 10. Synonymous variants may have different effects, including the alteration of splicing, impact on RNA stability, the binding of factors, and protein translation efficiency [19]. Thus, several tools were employed to study these aspects.

SpliceAI investigated possible splicing alteration or the creation of cryptic splice sites [14]. However, based on very low scores, no alteration of splicing was expected.

The mRNA’s secondary structure was assessed using the RNA fold web server [15]. From dot-bracket notation (Supplementary Figure S1), both sequences form similar secondary structures using minimum free energy (MFE) prediction, with consistent stem-loop domains, bulges, and hairpin structures. Both sequences maintain a conserved global topology using the centroid plots, with multiple stem-loop domains forming across the transcript. However, near position 800, where the synonymous mutation is located, we observed a slight difference in local base-pairing configurations and subtle changes in loop organization and pairing probabilities. These included minor shifts in hairpin formation stability, loop length, and the proximity of pairing in adjacent stems. These characteristics reflected a slightly lower ensemble diversity in the mutant, suggesting less structural variability, indicating that the RNA structure was more rigid or defined. The sequence with the 957A allele presented comparable MFE energy to the 957G allele and only slightly more negative centroid energy. Nevertheless, MFE energy is considered a more realistic prediction of RNA structure compared to the centroid one (Supplementary Table S1).

These local shifts were overall small, and they may not drastically alter the RNA function. However, they could affect local folding kinetics.

We used SplieAID [16] to investigate the possible creation or loss of consensus for RNA-binding proteins. This analysis proved that the two alleles may bind different RNA-binding factors. The wild-type 957G was recognized by ETR-3 and SRp30c, which are mainly involved in splicing, while the mutated 957A was identified by hnRNP H1 and hnRNP H2, which are mainly involved in pre-mRNA processing, mRNA metabolism, and transport.

Moreover, we accomplished miRNA Binding Site Prediction using miRDB [20]. The synonymous mutation did not significantly gain or lose miRNA binding sites, indicating a minimal impact on miRNA-mediated regulation.

Mutation Taster predicted the variant as deleterious, and the analysis showed high conservation at the nucleotide level, suggesting that this site is evolutionarily constrained [21].

### 3.4. ACADVL Synonymous Variants

By database and PubMed consultation, we retrieved six further synonymous variants in *ACADVL*, in addition to c.957G>A, p.Ser319= (Table 2). As expected, they were identified through confirmatory testing following positive results in newborn metabolic screening. An exception is represented by c.1317T>A, p.Gly439=, identified in an adult with liver dysfunction enrolled through the UK Biobank [22]. Interestingly, two of them—c.864C>T, p.Phe288= [9] and c.1077G>A, p.Ala359= [3,23,24]—were identified in homozygosity, both with supportive evidence of pathogenicity, and one—c.1077G>A, p.Ala359= —was found in a symptomatic newborn [24] (Table 2). Moreover, some of them are recurrent: (i) the c.957G>A, p.Ser319= was identified in three unrelated individuals, including the little girl reported here [9]. In two individuals, it was found in compound heterozygosity with c.848T>C, p.Val283Ala; in the third individual, it was found in compound heterozygosity with a splicing variant. (ii) c.1077G>A, p.Ala359= was identified in two unrelated individuals, either in homozygosis or in compound heterozygosity.

Furthermore, missense or nonsense pathogenic variants have been reported at codons Ser319, Ala359, Gly439, and Leu501.

### 3.5. Gene Expression Analysis

We analyzed RNA from the proband to explore these findings further.

**Figure 2:**
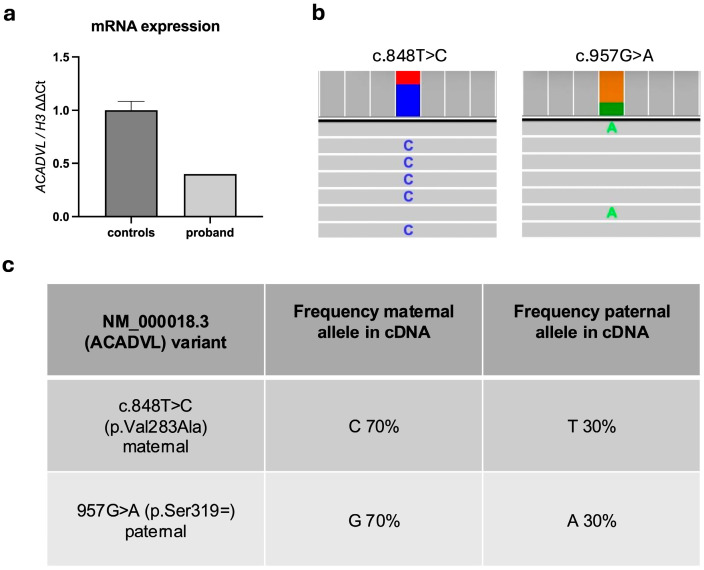
(**a**) *ACADVL* gene expression in controls and proband. (**b**) Allelic distribution from NGS sequencing; the two variants are shown, with histograms indicating allele frequency: blue—C; red—T; green—A; orange—G; example reads containing the C and A alleles are shown. (**c**) Allele frequency for the nucleotide position 848 and 957.

*ACADVL* gene expression on the proband’s blood cells revealed that *ACADVL* mRNA transcript levels were less than half compared to controls (Figure 2a). We observed no abnormal isoform production (i.e., exon-10 skipping) compared to healthy age-matched individuals, confirming the in silico prediction.

Moreover, allele expression analysis by NGS showed that the maternal allele was more highly expressed than the paternal allele (70% versus 30%), with no evidence of a cryptic splicing site (Figure 2b,c). These findings further supported the deleterious effect of the c.957G>A paternal variant on RNA stability.

## 4. Discussion

The introduction of metabolic newborn screening has facilitated the early detection of various metabolic diseases, accelerating diagnosis and treatment and significantly improving patient clinical management.

Very-long-chain acyl-CoA dehydrogenase deficiency (VLCADD) is one of the metabolic disorders included in Italian newborn screening. Its onset is variable, and its severity ranges widely [2].

Our study investigated a healthy child with biochemical alterations detected through the NBS.

Initial genetic analysis identified the child as a carrier of a variant in exon 9, c.848T>C, inherited from the mother, classified as pathogenic in clinical databases, including ClinVar and HGMD, and the literature. This variant has been reported in patients with homozygous or compound heterozygous status, associated with enzymatic activity between 3% and 27%. In contrast, heterozygous carriers typically exhibit residual enzymatic activity between 27% and 40% [9]. When detected in compound heterozygosity, this variant was associated with rhabdomyolysis, poor feeding, and altered liver function after the neonatal period [26]. The c.848T>C was localized in an outer loop, outside the binding and active sites of the protein; however, its presence altered the predicted protein 3d structure, potentially impacting protein functionality.

The proband here reported exhibited 19.8% residual enzymatic activity, a value that did not undoubtedly classify her as either a healthy carrier or an affected individual (mild form).

To further investigate, we conducted an in-depth analysis of the promoter and intronic regions, along with a re-evaluation of the coding sequence to identify potential variants of uncertain significance (VUS). This assessment revealed that the paternal allele carried a promoter variant at c-64T>C. In silico analyses predicted that it might disrupt the p300 transcription factor binding, potentially affecting gene expression. However, p300 primarily functions as a transcriptional co-activator or acetyltransferase [18] and the predicted binding region includes two mismatches in the p300 consensus motif. This variant presented a high allele frequency in the European population, and it was considered benign in the clinical database (ClinVar). Moreover, a 15-bp deletion spanning positions −64 to −78 has been observed in Danish VLCADD patients and healthy controls with a frequency of approximately 40% in both populations, suggesting that this region did not impact the RNA expression [27]. Therefore, this variant was unlikely to contribute to the RA observed.

A paternal synonymous variant, c.957G>A, was identified. This variant has been previously reported in compound heterozygosity either with c.848C>T or with c.1332+2T>A and was associated with slightly higher enzymatic activity (24 and 23% respectively) [9]. Conflicting classifications of pathogenicity were reported in the ClinVar database while HGMD classified it as a possible disease-causing mutation. Additionally, Mutation Taster predicted the variant as deleterious. While synonymous variants are often considered silent, they can have functional significance by affecting RNA splicing, stability, or structure [19].

The in silico analysis of potential splicing sites did not predict the creation of a cryptic splicing site at this localization. Furthermore, the functional analysis of exon 10 skipping showed no significant changes in isoform expression compared to controls.

The predicted secondary structures of the RNA containing the 957A and 957G alleles showed the same MFE structure, while the centroid structure showed subtle changes in loop organization and pairing probabilities. However, the MFE was almost comparable between the RNA containing the two alleles.

The analysis of potential binding factors revealed that the 957G allele was within a consensus sequence for two factors involved in splicing, ETR-3 [28] and SRp30c [29]. Moreover, ETR-3 played a role in mRNA stability. The mutated 957A allele was recognized by hnRNP H1 and hnRNP H2, two factors mainly involved in pre-mRNA processing, mRNA metabolism, and transport [30]. When comparing *ACADVL* mRNA levels between the proband and controls, a significant reduction was registered. Additionally, allele frequency analysis revealed a notable increase in the maternal allele carrying the pathogenic 848T variant. These findings indicated an overall reduction in *ACADVL* mRNA levels, where the allele carrying the pathogenic variant was also more present.

Given the promoter variant’s classification as benign, the 957A allele may affect RNA stability or disrupt the regulatory factors, ultimately influencing transcript levels. The 848C allele’s overrepresentation among *ACADVL* mRNA likely contributed to the observed decrease in VLCAD enzymatic activity. We, therefore, proposed an allelic imbalance in mRNA expression and a role of the c.957G>A as a pathogenic/hypomorphic allele.

This observation aligned with previous work by Hesse et al. [9], reporting the compound heterozygosity 848C/957A, although the authors did not explore the molecular implication in detail. Moreover, to our knowledge, this is the first report of a differential allelic expression involving the c.848T>C variant and *ACADVL*, too.

These considerations are further supported by a recent publication by Flowers et al., which focused exclusively on *ACADVL*. The authors proposed modifications to the standard guidelines of the American College of Medical Genetics and Genomics (ACMG) and the Association for Molecular Pathology (AMP) to better address the interpretive challenges specific to this complex disorder [8]. The new cut-off criteria based on GnomAD frequency set the BA1 criteria at ≥0.7% (benignity criteria stand-alone) and the PM2_pathogenicity supporting criteria at <0.1% [8]. These frequencies fit with the missense and synonymous genetic variants in the coding region (c.848T>C—0.1%, c.957G>A—0.03%), while the promoter variant (c.-64T>C) frequency is above the threshold (0.8%).

Based on the functional evidence reported here and the additional evidence retrieved (Table 2), we propose that c.957G>A, p.Ser319= be reclassified as a disease-causing variant.

Nevertheless, the reported case is still an asymptomatic girl. However, the possibility that the proband may develop VLCADD in the future—particularly in response to severe infection or metabolic stress—cannot be excluded.

Indeed, infectious agents can trigger acute metabolic derangements in hosts by increasing energy demands and compromising immune functionality, potentially exacerbating the course of inherited metabolic disorders [31]. Furthermore, late childhood, adolescent, and adult onset of VLCADD has been documented, often manifesting as recurrent episodes of rhabdomyolysis triggered by prolonged exercise or fasting [32]. A definitive treatment for VLCADD is not still available. However, nutrition prescriptions focusing on fat diet composition, avoiding prolonged fasting, the adaptation of effort levels (intensity, duration, and energy intake), and the treatment of fever at its onset are generally recommended to manage VLCADD [33]. Nevertheless, the long-term follow-up of patients revealed that this mild form had a good prognosis and often a reduction in rhabdomyolysis episodes [34].

Given the *ACADVL* compound heterozygosity, further evaluations are necessary to assess the potential risk of metabolic derangement.

Since 14% of the clinically relevant variants fall in non-coding regions (HGMD), comprehensive genetic sequencing and accurate variant annotation are crucial in order to define the prognosis and clinical management of patients with reduced enzymatic activity in the range of mildly affected VLCADD.

## Figures and Tables

**Table 1:** Details of the genetic findings in the proband. The dbSNP rs, gnomAD frequency, ClinVar and HGMD classification, and inheritance are reported for each variant.

NM_000018.3 ACADVL	Position	dbSNP rs	dbSNP Frequency European Population	gnomAD Genomes Frequency European Population	ClinVar Classification	HGMD Classification	Inheritance
c.-64T>C	Promoter	rs77051465	0.13	0.008	Benign	Not reported	Paternal
c.848T>C (p.Val283Ala)	Exon 9	rs113994167	0.002	0.001	Pathogenic	Disease-causing Mutation	Maternal
c.957G>A (p.Ser319=)	Exon 10	rs143870522	0.0001	0.00003	Conflicting classifications of pathogenicity	Possible disease-causing mutation (uncertain/less confident)	Paternal

**Table 2:** *ACADVL* synonymous variants including p.Ser319=.

NM_000018.3 *ACADVL* dbSNP rs	Position	gnomAD Total Frequency	ClinVar Classification	HGMD Classification	Supportive Evidence of Pathogenicity	Genotype	Study Population	Ref.
c.864C>T, p.Phe288= rs753748672	Exon 9	0.00001	LB	DM?	RA 31%	Homozygous	NBS	[9]
c.957G>A, p.Ser319= rs143870522	Exon 10	0.00003	Conflicting	DM?	RA 24%	Compound heterozygous with c.848T>C, p.Val283Ala, like in this study	NBS	[9]
					RA 23%	Compound heterozygous with c.1332+2T>A	NBS	[9]
c.1077G>A, p.Ala359= rs779458466	Exon 10	0.00001	Conflicting	DM	Predicted to affect splicing	Homozygous	NBS, symptomatic	[24]
						Compound heterozygous	NBS	[3]
						Heterozygous, de novo	ASD	[23]
c.1317T>A, p.Gly439= rs2142985210	Exon 13	0.000001	LB	DM	NR	Heterozygous	Individual from the UK Biobank with liver dysfunction	[22]
c.1464C>T, p.Gly488=	Exon 15	-	-	DM?	PM2+PP4	Compound heterozygous with c.1795G>A, p.E599K	NBS in China, symtomatic	[25]
c.1501C>T, p.Leu501	Exon 15	-	-	DM	Predicted to affect splicing (missplicing)	Compound heterozygous with c.865G>A, p.G289R	NBS	[26]
c.1617T>C, p.Ala539= rs1555528948	Exon 17	-	VUS	DM	Predicted to affect splicing by Mutation Taster	Compound heterozygous with c.1708_1717GACGGGGCCA	NBS, symptomatic	[26]

LB, likely benign; DM, disease-causing variant; VUS, variant of uncertain significance; NBS, newborn screening; ASD, autism spectrum disorder; NR, not reported; PM2, Pathogenic Moderate 2: provides moderate evidence based on allele frequency in population databases; PP4, Phenotype Specific 4: offers supporting evidence based on the patient’s phenotype being consistent with a specific genetic etiology; RA, residual VLCAD activity; -, not available.

## Data Availability

The original contributions presented in this study are included in the article/Supplementary Materials. Further inquiries can be directed to the corresponding author.

## Supplementary Materials

The following supporting information can be downloaded at: https://www.mdpi.com/article/10.3390/genes16050538/s1, Figure S1: Centroid structure of the RNA containing the containing c.957 G (wild-type) and A allele; Figure S2: comparison of centroid RNA secondary structures for the synonymous and wild-type mRNA sequences; Table S1: Acylcarnitine profile as measured at the proband’s follow-up visit; Table S2: Minimum Free Energy (MFE) and Stability Comparison between the RNA containing c.957 A a(wild-type) and G allele (mutant).
