# Supplementary material for: ACADVL Deep Sequencing in a Case Study: Beyond the Common c.848T>C Pathogenic Variant
Source: Genes (Basel). 2025 Apr 30;16(5):538. doi: 10.3390/genes16050538 (PMC12111055; doi:10.3390/genes16050538)

Supplementary Table S1. Acylcarnitine profile as measured at the proband's follow-up visit.

Acylcarnitine in µmol/L (reference interval)	14 months	21 months
**Acetyl carnitine, C2** (3.78-23.67)	NA	24.08
**Dodecanoylcarnitine, C12** (0.01-0.18)	0.38	0.31
**Dodecenoylcarnitine, C12:1** (0.01-0.11)	0.20	0.20
**Tetradecanoylcarnitine or myristoylcarnitine, C14** (0.01-0.07)	0.14	0.15
**Tetradecenoylcarnitine, C14:1** (0.02-0.17)	0.51	0.61
**Tetradecadienoylcarnitine, C14:2** (0.00-0.04)	0.10	0.10
**Hexadecenoylcarnitine, C16:1** (0.01-0.04)	0.05	0.08

NA, not available

Supplementary Table S2. Minimum Free Energy (MFE) and Stability Comparison between the RNA containing c.957 A (wild-type) and G allele (mutant)

Metric	Wild-Type	Mutant
MFE Structure Energy	-819.50 kcal/mol	-820.30 kcal/mol
Thermodynamic Ensemble Free Energy	-851.17 kcal/mol	-851.80 kcal/mol
Centroid Structure Free Energy	-582.20 kcal/mol	-610.30 kcal/mol
Ensemble Diversity	630.92	576.07

Supplementary Figure S1.

Centroid structure of the RNA containing the containing c.957 G (wild-type) and A allele.

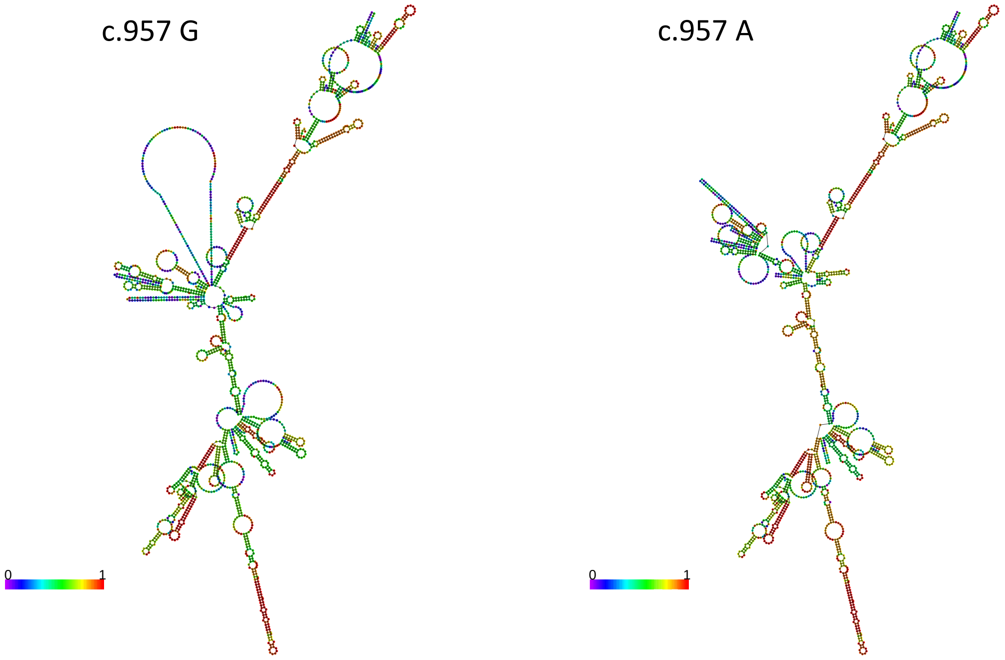

Minimum Free Energy (MFE) structure of the RNA containing the containing c.957 G (wild-type) and A allele

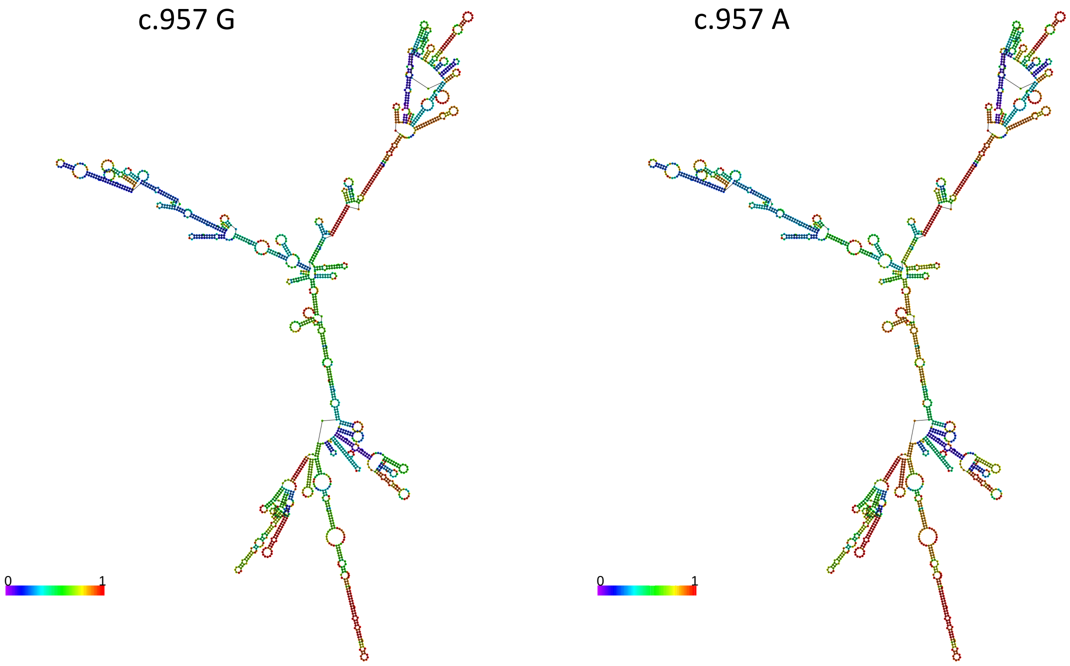

Supplementary Figure S2

Zoomed-in comparison of centroid RNA secondary structures for the synonymous and wild-type mRNA sequences surrounding the mutation site (nt ~799).
Base-pairing patterns are represented using dot-bracket notation, with (indicating base-pair opening (blue),) for closing (red), and . representing unpaired nucleotides (gray).
The structural arrangement remains largely conserved between wild-type and mutant sequences, with minimal deviation in pairing configuration, supporting the interpretation of a subtle yet potentially functionally relevant local stabilization effect.

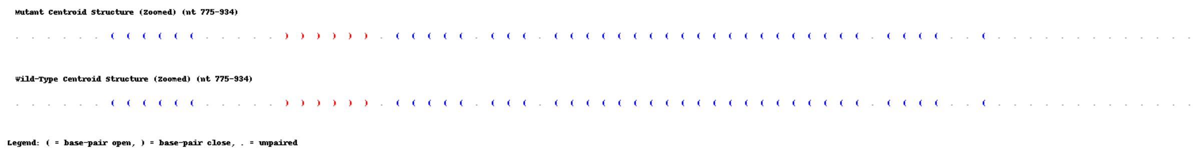

Supplement: Supplementary file 1 [file genes-16-00538-s001.zip › genes-3608965-supplementary.pdf]
